# Adult Day Services and COVID: A Crisis in Ohio

**DOI:** 10.1093/geroni/igab046.423

**Published:** 2021-12-17

**Authors:** Holly Dabelko-Schoeny, Susan Wallace, Salli Bolin

**Affiliations:** 1 The Ohio State University, The Ohio State University, Ohio, United States; 2 LeadingAge Ohio, Columbus, Ohio, United States; 3 MemoryLane Care Services, Toledo, Ohio, United States

## Abstract

An Ohio Executive Order forced adult day service providers across the state to close from March 24, 2020 until September 21, 2020 due to COVID, resulting in significant hardship for providers and families. In fact, 65% of programs reported laying off or reducing staff and 83% of directors reported participants had to move to higher and more expensive levels of care such as nursing homes and assisted living. Programs reported that 74% of caregivers had to choose between working and taking care of their family members. Ninety-one percent of ADS program directors in Ohio reported their caregivers were experiencing an increase in stress and anxiety. This paper explores the experiences of Ohio adult day providers during the COVID epidemic, and identifies the challenges and opportunities to coalition building to educate policy makers about day services and the crucial care centers provide.

